# Improving isobutanol production with the yeast *Saccharomyces cerevisiae* by successively blocking competing metabolic pathways as well as ethanol and glycerol formation

**DOI:** 10.1186/s13068-019-1486-8

**Published:** 2019-07-02

**Authors:** Johannes Wess, Martin Brinek, Eckhard Boles

**Affiliations:** Institute of Molecular Biosciences, Goethe University Frankfurt, Max-von-Laue-Str. 9, 60438 Frankfurt am Main, Germany

**Keywords:** Biofuel, Isobutanol, Valine degradation, Ehrlich pathway, Fermentation, Ethanol, Glycerol, NADH/NADPH redox cofactor imbalance, *Saccharomyces cerevisiae*

## Abstract

**Background:**

Isobutanol is a promising candidate as second-generation biofuel and has several advantages compared to bioethanol. Another benefit of isobutanol is that it is already formed as a by-product in fermentations with the yeast *Saccharomyces cerevisiae*, although only in very small amounts. Isobutanol formation results from valine degradation in the cytosol via the Ehrlich pathway. In contrast, valine is synthesized from pyruvate in mitochondria. This spatial separation into two different cell compartments is one of the limiting factors for higher isobutanol production in yeast. Furthermore, some intermediate metabolites are also substrates for various isobutanol competing pathways, reducing the metabolic flux toward isobutanol production. We hypothesized that a relocation of all enzymes involved in anabolic and catabolic reactions of valine metabolism in only one cell compartment, the cytosol, in combination with blocking non-essential isobutanol competing pathways will increase isobutanol production in yeast.

**Results:**

Here, we overexpressed the three endogenous enzymes acetolactate synthase (Ilv2), acetohydroxyacid reductoisomerase (Ilv5) and dihydroxy-acid dehydratase (Ilv3) of the valine synthesis pathway in the cytosol and blocked the first step of mitochondrial valine synthesis by disrupting endogenous *ILV2*, leading to a 22-fold increase of isobutanol production up to 0.22 g/L (5.28 mg/g glucose) with aerobic shake flask cultures. Then, we successively deleted essential genes of competing pathways for synthesis of 2,3-butanediol (*BDH1* and *BDH2*), leucine (*LEU4* and *LEU9*), pantothenate (*ECM31*) and isoleucine (*ILV1*) resulting in an optimized metabolic flux toward isobutanol and titers of up to 0.56 g/L (13.54 mg/g glucose). Reducing ethanol formation by deletion of the *ADH1* gene encoding the major alcohol dehydrogenase did not result in further increased isobutanol production, but in strongly enhanced glycerol formation. Nevertheless, deletion of glycerol-3-phosphate dehydrogenase genes *GPD1* and *GPD2* prevented formation of glycerol and increased isobutanol production up to 1.32 g/L. Finally, additional deletion of aldehyde dehydrogenase gene *ALD6* reduced the synthesis of the by-product isobutyrate, thereby further increasing isobutanol production up to 2.09 g/L with a yield of 59.55 mg/g glucose, corresponding to a more than 200-fold increase compared to the wild type.

**Conclusions:**

By overexpressing a cytosolic isobutanol synthesis pathway and by blocking non-essential isobutanol competing pathways, we could achieve isobutanol production with a yield of 59.55 mg/g glucose, which is the highest yield ever obtained with *S. cerevisiae* in shake flask cultures. Nevertheless, our results indicate a still limiting capacity of the isobutanol synthesis pathway itself.

## Background

Isobutanol is a promising candidate for second-generation biofuels which can be produced from biomass via fermentative microbial processes. Compared to the currently most common first-generation biofuel ethanol, isobutanol has several advantages such as a higher combustion power, a reduced aqueous miscibility and a weaker corrosivity [36]. Moreover, because of its physical and chemical properties, isobutanol is compatible with current pipe systems and processes of the gasoline industry without the need for infrastructural adaption. Another benefit is that isobutanol is already produced as a by-product in fermentations with the yeast *S. cerevisiae*, although only in very small amounts [5, 19].

**Fig. 1:**
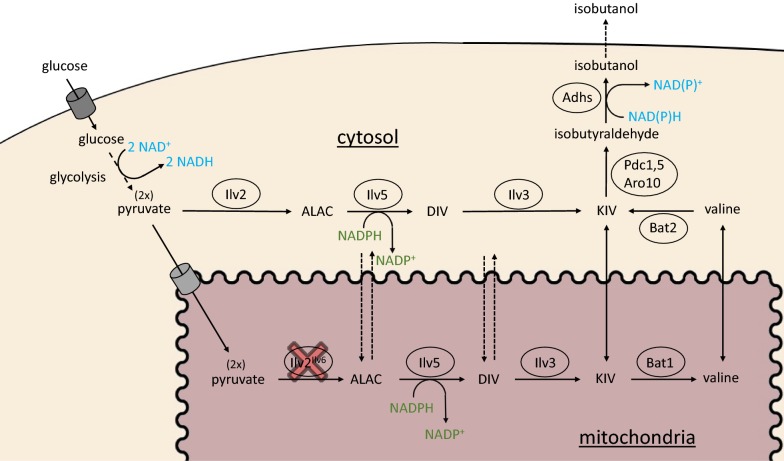
Isobutanol biosynthesis pathway in yeast *S. cerevisiae*. ALAC: 2-acetolactate; DIV: 2,3-dihydroxyisovalerate; KIV: 2-ketoisovalerate. Glucose is converted to two molecules of pyruvate in glycolysis, generating two molecules of NADH. In the native pathway, after transport into mitochondria, two molecules of pyruvate are condensed to one molecule of 2-acetolactate (ALAC) by the Ilv2^Ilv6^ complex. Reduction and isomerization of ALAC to 2,3-dihydroxyisovalerate (DIV) by Ilv5 requires redox cofactor NADPH. DIV is dehydrated to 2-ketoisovalerate (KIV) by Ilv3. KIV is either directly released into the cytosol or converted to valine in mitochondria by Bat1. After export into the cytosol, valine is degraded via the Ehrlich pathway to isobutanol, starting with a transamination to KIV by Bat2. KIV is decarboxylated to isobutyraldehyde by Pdc1, 5 and Aro10. Finally, isobutyraldehyde is reduced to isobutanol, generating one molecule of NAD(P)^+^. By transformation of the episomal 2µ-plasmid IsoV100, we overexpressed Ilv2, Ilv5 and Ilv3 in the cytosol. Simultaneous deletion of mitochondrial Ilv2 resulted in a cytosolic isobutanol pathway mixed with part of a mitochondrial pathway

Isobutanol is synthesized in the yeast cytosol by a three-step degradation of the amino acid valine via the Ehrlich pathway (Fig. 1) [8, 19]. In contrast, biosynthesis of valine from pyruvate occurs exclusively in mitochondria. After entering mitochondria, two molecules of pyruvate are condensed to 2-acetolactate (ALAC) by acetolactate synthase Ilv2, feedback regulated by its subunit Ilv6 [32]. ALAC is reduced and isomerized to 2,3-dihydroxyisovalerate (DIV) by acetohydroxyacid reductoisomerase Ilv5. For this reaction, NADPH is used as redox cofactor and oxidized to NADP^+^. The dihydroxy-acid dehydratase Ilv3 dehydrates DIV to 2-ketoisovalerate (KIV). KIV can either be directly converted to valine by the branched-chain amino acid aminotransferase Bat1 in mitochondria, or first be exported into the cytosol to be processed to valine by the branched-chain amino acid transaminase Bat2 [22]. For further conversion to isobutanol, valine must be transaminated back to KIV by Bat2 or KIV can be used directly. After the decarboxylation of KIV by pyruvate decarboxylases 1, 5 (and 6) (Pdc1, Pdc5, Pdc6) and phenylpyruvate decarboxylase Aro10, isobutyraldehyde is reduced to isobutanol by alcohol dehydrogenases 1–5 (Adh1-5), oxidizing one molecule of redox cofactor NADH to NAD^+^ [3, 5, 6, 19]. Adh6 might also be involved in this reaction to regenerate the redox cofactor NADP^+^ [25]. Finally, isobutanol diffuses out of the cell.

**Fig. 2:**
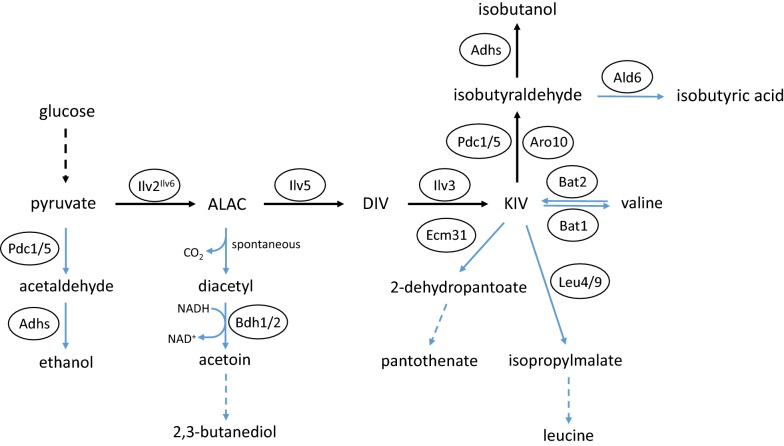
Isobutanol competing pathways in yeast *S. cerevisiae*. ALAC: 2-acetolactate; DIV: 2,3-dihydroxyisovalerate; KIV: 2-ketoisovalerate

**Fig. 3:**
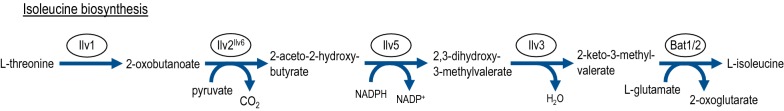
Isoleucine biosynthesis pathway in yeast *S. cerevisiae*

Within the metabolic pathway to isobutanol, several intermediate metabolites, such as pyruvate, ALAC, KIV and isobutyraldehyde, also serve as substrates for various other biosyntheses (Fig. 2). Under anaerobic conditions and under high glucose concentrations (Crabtree effect), pyruvate undergoes fermentation to ethanol, which is the most competing pathway. There, pyruvate is decarboxylated to acetaldehyde by Pdc1 and Pdc5 (and Pdc6), and further reduced to ethanol by Adh1 and other Adhs. Another competing pathway is the formation of 2,3-butanediol from ALAC. After a spontaneous decarboxylation of ALAC, diacetyl is oxidized to acetoin by NAD-dependent butanediol dehydrogenases Bdh1 and Bdh2 and further reduced to 2,3-butanediol by Bdh1 [16, 26]. Furthermore, besides valine synthesis and the Ehrlich pathway, KIV is also a substrate for biosyntheses of pantothenate and leucine. In the first step of pantothenate biosynthesis, KIV is converted to 2-dehydropantoate by 3-methyl-2-oxobutanoate hydroxymethyltransferase Ecm31. Within leucine biosynthesis, 2-isopropylmalate synthases Leu4 and Leu9 catalyze the first step from KIV to 2-isopropylmalate. In the last step of valine degradation, isobutyraldehyde can either be converted to isobutanol by various Adhs or oxidized to isobutyric acid by aldehyde dehydrogenases like Ald6. Within the isoleucine biosynthesis pathway, the Ilv2^Ilv6^ enzyme complex condenses pyruvate and 2-oxobutanoate to form 2-aceto-2-hydroxybutyrate (Fig. 3). Additionally, the intermediates 2-aceto-2-hydroxybutyrate and 2,3-dihydroxy-3-methylvalerate are processed by the enzymes Ilv5 and Ilv3, respectively, which also play a major role in the isobutanol pathway (Fig. 3). Thus, ALAC and 2-aceto-2-hydroxybutyrate as well as DIV and 2,3-dihydroxy-3-methylvalerate might compete for accessibility to Ilv5 and Ilv3, respectively. Hence, the isoleucine biosynthesis cannot only be considered as a competing pathway, but also as a competitive inhibitory pathway for isobutanol production. Taken together, these competing pathways reduce the availability of metabolites for isobutanol biosynthesis and therefore decrease the metabolic flux toward isobutanol production and isobutanol yields.

In summary, spatial separation of anabolic and catabolic reactions of valine metabolism to mitochondria and cytosol, respectively, as well as competitions between the isobutanol pathway and other pathways for intermediate metabolites or enzymes might be limiting factors for high synthesis levels of isobutanol in *S. cerevisiae*. Two different strategies are pursued to overcome the challenges of pathway compartmentalization. The first approach addresses the relocalization of the cytosolic Ehrlich pathway enzymes into mitochondria [1, 18, 37], together with the native valine synthesis pathway. The second approach is based on the relocalization of the mitochondrial valine synthesis enzymes into the cytosol, together with the native Ehrlich pathway enzymes [3]. In this previous work, Brat et al. expressed N-terminally truncated, codon-optimized isoforms of Ilv2, Ilv3 and Ilv5 in the cytosol while simultaneously blocking the first step of mitochondrial valine synthesis by disruption of endogenous *ILV2*, but leaving additional copies of mitochondrially located Ilv5 and Ilv3.

We hypothesized that additional blocking of non-essential, competing pathways by deletions of genes encoding key enzymes of competing pathways should increase the amount of intermediate metabolites available for isobutanol biosynthesis and thereby force the metabolic flux toward isobutanol production as it has already been shown before for individual cases (reviewed in [24]). Furthermore, since Ilv2, Ilv3 and Ilv5 are key enzymes of the valine biosynthesis, blocking the isoleucine biosynthesis pathway might also improve enzyme kinetics of Ilv2, Ilv3 and Ilv5 for the desired reactions and by this further increase isobutanol production.

Here, we expressed the cytosolic isobutanol pathway as published by Brat et al. [3] and successively blocked all unproductive competing pathways by gene deletions. With this strategy we could successfully increase the isobutanol production up to 2.09 g/L with a yield of 59.55 mg/g glucose, corresponding to a more than 200-fold increase compared to the wild type (wt).

## Results and discussion

### Isobutanol pathway engineering and block of non-essential isobutanol competing pathways

**Fig. 4:**
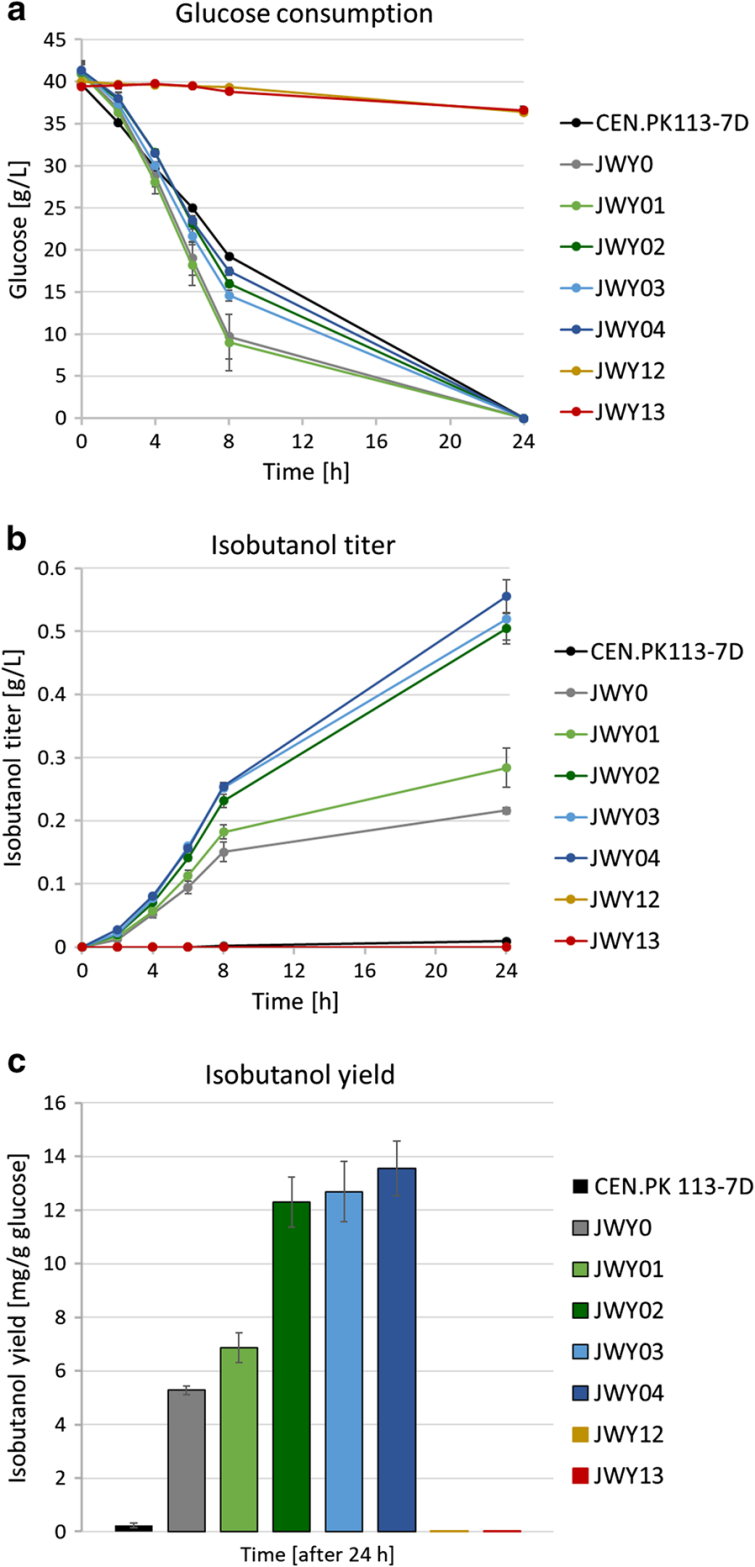
Improvements of isobutanol production by blocking competing pathways in *S. cerevisiae*. Fermentation experiments were performed aerobically at 30 °C in shake flasks in selective SCD medium without valine containing 40 g/L glucose. Pre-cultures carrying the episomal 2µ-plasmid IsoV100 were grown aerobically in fermentation medium, harvested at an OD_600_ ≤ 3 and inoculated in fresh fermentation medium at a final OD_600_ of 8. Fermentations were performed in duplicate per experiment and repeated at least twice. Error bars indicate standard deviation for experiments performed twice and standard error for experiments performed at least three times, respectively. **a** Glucose consumption after successively blocking of competing isobutanol pathways by deletion of key enzymes of biosynthesis pathways: CEN.PK113-7D (wt); JWY0 (*∆ilv2)*; JWY01 (*∆ilv2; Δbdh1*; *Δbdh2*); JWY02 (*∆ilv2*; *Δbdh1*; *Δbdh2*; *Δleu4*; *Δleu9*); JWY03 (*∆ilv2*; *Δbdh1*; *Δbdh2*; *Δleu4*; *Δleu9*; *Δecm31*); JWY04 (*∆ilv2*; *Δbdh1*; *Δbdh2*; *Δleu4*; *Δleu9*; *Δecm31*; *Δilv1*); JWY12 (*∆ilv2*; *Δbdh1*; *Δbdh2*; *Δleu4*; *Δleu9*; *Δecm31*; *Δilv1*; *Δpdc1*::*MTH1*; *Δpdc5*); JWY13 (*∆ilv2*; *Δbdh1*; *Δbdh2*; *Δleu4*; *Δleu9*; *Δecm31*; *Δilv1*; *Δpdc1*; *Δpdc5*; *Δmth1* (+ 169; + 393). **b** Isobutanol titer. **c** Isobutanol yield after 24 h

To localize the isobutanol biosynthetic pathway into the cytosol, all strains were transformed with the episomal 2µ-plasmid IsoV100 [3], expressing codon-optimized, N-terminally shortened versions of Ilv2 (Ilv2Δ54), Ilv5 (Ilv5Δ48) and Ilv3 (Ilv3Δ19), lacking the mitochondrial targeting sequences to prevent import of Ilv2, Ilv5 and Ilv3 into mitochondria. Additionally, the native *ILV2* gene encoding the mitochondrial Ilv2 enzyme was deleted as it has been shown that this deletion massively increases isobutanol production by a still unknown mechanism ([3] and see results below (Fig. 4b, c)). Since the mitochondrial membrane is probably permeable for ALAC, DIV and KIV [3], the mitochondrial versions of Ilv5 and Ilv3 were not eliminated and might additionally contribute to the production of KIV. In our previous work the highest isobutanol yields were obtained with strains additionally expressing the mitochondrial versions of Ilv5 and Ilv3 [3]. Thus, our isobutanol pathway should be defined as a cytosolic pathway mixed with part of a mitochondrial pathway.

In addition to the expression of the cytosolic isobutanol pathway, non-essential competing pathways of the isobutanol biosynthesis were blocked to force the metabolic flux toward isobutanol synthesis. Starting from wt strain CEN.PK113-7D with a blocked mitochondrial valine biosynthesis resulting from an *ilv2* deletion (JWY0), we successively deleted the following competing pathways by using the CRISPR-Cas9 system: by deleting *BDH1* and *BDH2* (strain JWY01), we interrupted 2,3-butanediol biosynthesis after the spontaneous diacetyl formation step. Next, we blocked the first catalytic reaction of the leucine biosynthesis pathway by deleting *LEU4* and *LEU9*, resulting in strain JWY02. By the deletion of *ECM31* (strain JWY03), the pantothenate biosynthesis pathway was blocked. To prevent competitive outflow of pyruvate into isoleucine biosynthesis and to increase the accessibility of ALAC and DIV to Ilv5 and Ilv3, respectively, we interrupted the isoleucine biosynthesis pathway by deletion of *ILV1*, resulting in strain JWY04. Valine biosynthesis was inhibited by deletion of *BAT1* and *BAT2* (strain JWY07). Since *PDC6* normally is not expressed [20], deletions of *PDC1* together with *PDC5* were sufficient to block ethanol biosynthesis. However, *pdc*^−^ strains cannot grow on high glucose concentrations [31]. To restore growth on glucose of Pdc-deficient strains, in strain JWY12 the *PDC1* coding region was replaced by *MTH1*, thereby placing *MTH1* under control of the strong *PDC1* promoter, and in strain JWY13 an internal deletion in *MTH1* (*MTH1∆T*) was engineered which stabilizes the Mth1 protein [31]. Both approaches should increase the amount of Mth1 protein and thereby reduce glucose transport and restore (slow) consumption and growth of Pdc-deficient cells. However, in contrast to JWY12 only strain JWY13 could slowly grow on agar plates with 2% (w/v) glucose as carbon source (data not shown).

### Elimination of competing pathways improves isobutanol production

To investigate the effects of the lack of competing pathways and of the relocation of all enzymes in the cytosol on isobutanol production, aerobic shake flask fermentations were performed. Therefore, transformants were inoculated as pre-cultures in shake flasks in SCD media without valine. Omission of valine was shown to increase isobutanol production for an unknown reason [3]. We could repeatedly confirm this effect (data not shown, and see results below with *bat1* and *bat2* deletion mutants). Metabolite analyses showed a low isobutanol titer of 0.01 g/L (0.23 mg/g glucose) after 24 h for the wt strain CEN.PK113-7D expressing the cytosolic isoforms of Ilv2, Ilv5, and Ilv3 (Fig. 4b, c). Blocking of the mitochondrial valine biosynthesis by deletion of *ILV2* encoding the mitochondrial form of acetolactate synthase (strain JWY0) led to a major increase of the isobutanol titer up to 0.22 g/L (5.28 mg/g glucose) as it has already been shown by Brat et al. [3]. Inhibition of the 2,3-butanediol biosynthesis pathway by the deletion of *BDH1/2* (strain JWY01) further increased isobutanol production to a titer of 0.28 g/L (6.86 mg/g glucose). This effect might not only be explained by preventing the withdrawal of ALAC from isobutanol synthesis (as just diacetyl should be accumulating instead of 2,3-butanediol), but also by preventing the consumption of NADH by the Bdh reactions which then can be used instead to force the final reduction of isobutyraldehyde to isobutanol by Adhs.

Further suppression of leucine biosynthesis by deletion of *LEU4/9* (strain JWY02) had an additional large effect on isobutanol levels and resulted in a titer of 0.50 g/L (12.31 mg/g glucose). Further blocking of pantothenate biosynthesis by deletion of *ECM31* (strain JWY03) had no statistically significant effect on isobutanol production (0.52 g/L, 12.69 mg/g glucose). Maximum isobutanol titers of 0.56 g/L (13.54 mg/g glucose) were reached with strain JWY04 after interrupting the isoleucine biosynthesis pathway by deletion of *ILV1*. These results are in good agreement with similar previous approaches [21]. Since it was necessary to inoculate the pre-cultures in SCD media without valine, Bat1/2-deficient strain JWY07 with its blocked valine biosynthesis was not able to grow to a sufficient OD for fermentation experiments. However, even slight reductions of the valine synthesis by deleting only *BAT1* (JWY05) or *BAT2* (JWY06) had negative effects on growth in media without valine and on isobutanol production, in contrast to other work [34]. Although the addition of valine to the medium improved the growth of the strains, it resulted in sharp decreases in isobutanol production. These observations are in accordance with the findings of Brat et al. [3] concerning a negative effect on isobutanol synthesis by addition of valine.

Therefore, we did not further consider deletions of the transaminases and continued with the suppression of ethanol formation.

### Deletion of *PDC* genes has a negative effect on isobutanol production

Additional deletions of *PDC1* and *PDC5*, even after restoring slow growth by overexpressing Mth1 (strain JWY12) or by engineering Mth1∆T (strain JWY13), had negative effects and stopped isobutanol biosynthesis completely (Fig. 4b, c). Even after 168 h, isobutanol was not detectable (data not shown). This confirmed the results of Choo et al. [4], Brat et al. [3] and Milne et al. [28], indicating that Pdc1 and Pdc5 significantly contribute also in the conversion of KIV to isobutyraldehyde within the Ehrlich pathway. Moreover, while glucose was consumed completely within 24 h in strains JWY0 to JWY04, *pdc1/5* double deletions severely affected the glucose consumption even with overexpressed or engineered Mth1ΔT (glucose consumption after 168 h: 7.54 g/L for JWY12 and 6.79 g/L for JWY13) (Fig. 4a). An explanation for this unexpected low glucose consumption and isobutanol production might be an arising C_2_ auxotrophy in pdc^−^ cells, as Pdcs provide C_2_ compounds for synthesis of cytosolic acetyl-coenzyme A [10, 11, 35]. To investigate if C_2_ auxotrophy was the limiting factor in Pdc-deficient cells, fermentation experiments were repeated with a supplementation of 1% (v/v) ethanol as C_2_ compound source to the fermentation media to compensate the lack of C_2_ sources.

**Fig. 5:**
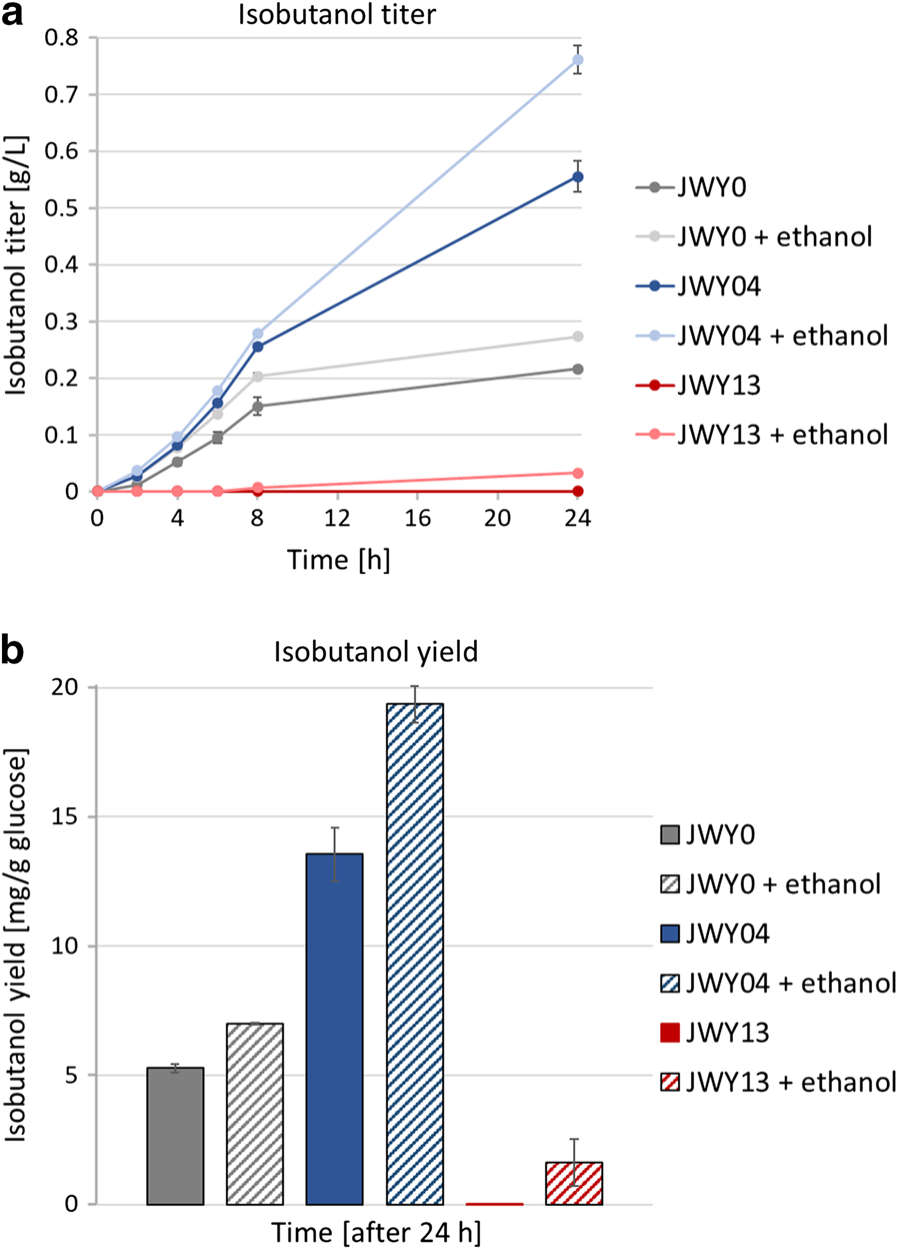
Supplementation of ethanol increases isobutanol production in *S. cerevisiae*. Fermentation experiments were performed aerobically at 30 °C in shake flasks in selective SCD medium without valine containing 40 g/L glucose. Pre-cultures carrying the episomal 2µ-plasmid IsoV100 were grown aerobically in fermentation medium, harvested at an OD_600_ ≤ 3 and inoculated in fresh fermentation medium at a final OD_600_ of 8. Fermentations were performed in duplicate per experiment and repeated at least twice. Error bars indicate standard deviation for experiments performed twice and standard error for experiments performed at least three times, respectively. **a** Isobutanol titer after successively blocking of competing pathways by deletion of key enzymes of biosynthesis pathways and supplementation of 1% (v/v) ethanol to fermentation medium: JWY0 (*∆ilv2*); JWY04 (*∆ilv2*; *Δbdh1*; *Δbdh2*; *Δleu4*; *Δleu9*; *Δecm31*; *Δilv1*); JWY13 (*∆ilv2*; *Δbdh1*; *Δbdh2*; *Δleu4*; *Δleu9*; *Δecm31*; *Δilv1*; *Δpdc1*; *Δpdc5*; *Δmth1* (+ 169; + 393). **b** Isobutanol yield after 24 h

Results showed that supplementation of ethanol increased isobutanol titer only slightly to 0.03 g/L (1.62 mg/g glucose) in JWY13 (Fig. 5a, b). Moreover, this improvement was not exclusively restricted to Pdc-deficient strain JWY13, but could also be observed in the Pdc-proficient strains JWY0 and JWY04, increasing the isobutanol titer from 0.22 to 0.27 g/L (7.0 mg/g glucose) and from 0.56 to 0.76 g/L (19.36 mg/g glucose), respectively. This clearly demonstrates that a potential C_2_ auxotrophy is not the bottleneck for isobutanol production in Pdc-deficient cells. The increased isobutanol production after ethanol addition to all the tested strains might arise from an unknown positive effect of ethanol on glucose sensing, from the higher total amount of available carbon sources or from effects on the redox balance via its NAD^+^- and NADP^+^-dependent oxidation to acetate (see below).

**Fig. 6:**
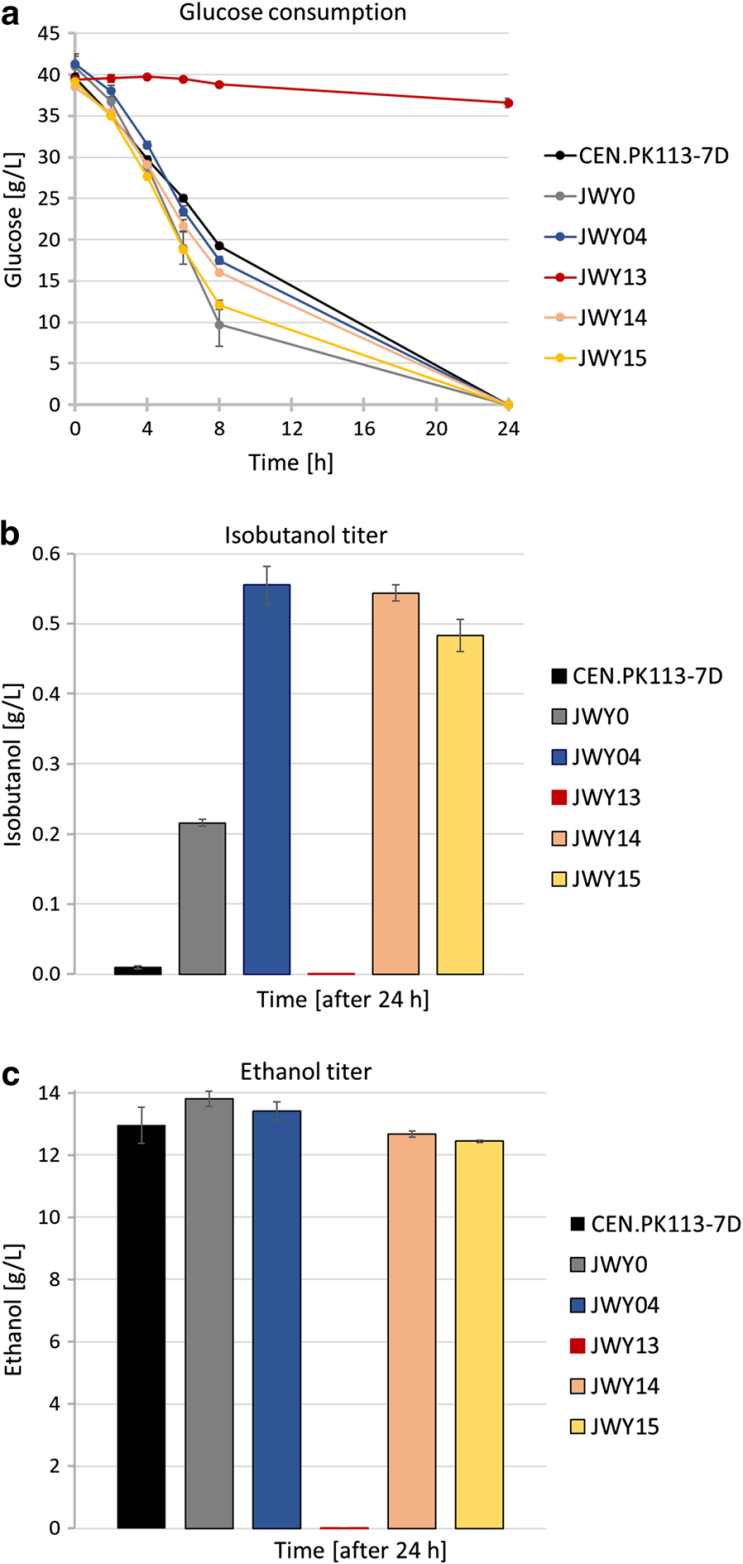
Isobutanol production was not improved by single deletions of *PDC1* or *PDC5*. Fermentation experiments were performed aerobically at 30 °C in shake flasks in selective SCD medium without valine containing 40 g/L glucose. Pre-cultures carrying the episomal 2µ-plasmid IsoV100 were grown aerobically in fermentation medium, harvested at an OD_600_ ≤ 3 and inoculated in fresh fermentation medium at a final OD_600_ of 8. Fermentations were performed in duplicate per experiment and repeated at least twice. Error bars indicate standard deviation for experiments performed twice and standard error for experiments performed at least three times, respectively. **a** Glucose consumption after successively blocking of competing isobutanol pathways by deletion of key enzymes of biosynthesis pathways: JWY0 (*∆ilv2*); JWY04 (*∆ilv2*; *Δbdh1*; *Δbdh2*; *Δleu4*; *Δleu9*; *Δecm31*; *Δilv1*); JWY13 (*∆ilv2*; *Δbdh1*; *Δbdh2*; *Δleu4*; *Δleu9*; *Δecm31*; *Δilv1*; *Δpdc1*; *Δpdc5*; *Δmth1* (+ 169; + 393); JWY14 (*∆ilv2*; *Δbdh1*; *Δbdh2*; *Δleu4*; *Δleu9*; *Δecm31*; *Δilv1*; *Δpdc1*); JWY15 (*∆ilv2*; *Δbdh1*; *Δbdh2*; *Δleu4*; *Δleu9*; *Δecm31*; *Δilv1*; *Δpdc5*). **b** Isobutanol titer after 24 h. **c** Ethanol production after 24 h

Due to an autoregulation of *PDC* expression, *pdc1* and *pdc5* single mutants exhibit only slightly reduced Pdc activities [7, 29]. Thus, we hypothesized that a single deletion of *PDC1* or *PDC5* in the JWY04 strain background will have minor effects on ethanol formation, sustain most of the glycolytic flux but increase the availability of pyruvate for the isobutanol pathway [23]. Additionally, due to the contribution of Pdcs in the isobutyraldehyde forming reaction from KIV within the Ehrlich pathway, single deletions should lead to minor effects on isobutanol production. Therefore, we deleted *PDC1* (strain JWY14) or *PDC5* (JWY15) individually and performed fermentations. Indeed, glucose consumption of JWY14 and JWY15 was comparable to the Pdc-proficient strains, consuming glucose completely within 24 h (Fig. 6a). Compared to the wt strain CEN.PK113-7D (13 g/L), JWY0 (13.8 g/L) and JWY04 (13.4 g/L), single deletion strains JWY14 (*∆pdc1*) and JWY15 (*∆pdc5*) showed only minor effects in ethanol production, resulting in ethanol titers of 12.6 g/L and 12.3 g/L, respectively, whereas the *pdc1/5* double deletion strain JWY13 showed an ethanol titer of only 0.01 g/L (Fig. 6c). But, in contrast to our expectations, JWY14 and JWY15 showed even slightly reduced isobutanol titers of 0.54 g/L and 0.48 g/L, respectively (Fig. 6b). Although it is described in the literature that a single deletion of *PDC1* or *PDC5* improves isobutanol production significantly [23, 26], this could not be observed with our strategy and strain background.

The results indicate that either the metabolic flux toward isobutanol has already reached its maximum due to limitations in one or more enzyme reactions or that an unbalanced redox cofactor situation—not enough NADPH for Ilv5, not enough regeneration of NAD^+^ by isobutyraldehyde reduction—prevents further increases in isobutanol production.

### Reduction of ethanol, glycerol and isobutyric acid formation enhances isobutanol production

**Fig. 7:**
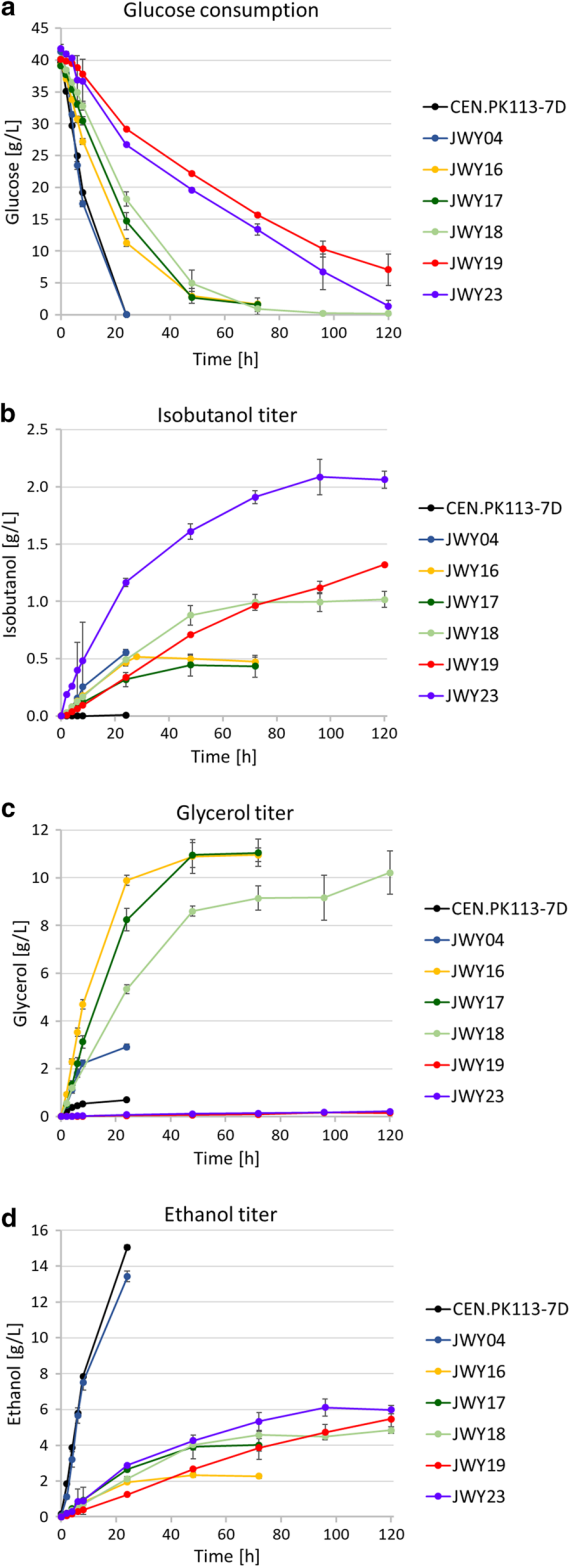
Reduction of ethanol, glycerol and isobutyric acid biosyntheses increases isobutanol production. Fermentation experiments were performed aerobically at 30 °C in shake flasks in selective SCD medium without valine containing 40 g/L glucose. Pre-cultures carrying the episomal 2µ-plasmid IsoV100 were grown aerobically in fermentation medium, harvested at an OD_600_ ≤ 3 and inoculated in fresh fermentation medium at a final OD_600_ of 8. Fermentation experiments were performed at least in duplicate. Error bars indicate standard deviation for experiments performed twice and standard error for experiments performed at least three times, respectively. **a** Glucose consumption after successively blocking of competing isobutanol pathways by deletion of key enzymes of biosynthesis pathways: CEN.PK113-7D (wt); JWY04 (*∆ilv2*; *Δbdh1*; *Δbdh2*; *Δleu4*; *Δleu9*; *Δecm31*; *Δilv1*); JWY16 (*∆ilv2*; *Δbdh1*; *Δbdh2*; *Δleu4*; *Δleu9*; *Δecm31*; *Δilv1*; *Δadh1*); JWY17 (*∆ilv2*; *Δbdh1*; *Δbdh2*; *Δleu4*; *Δleu9*; *Δecm31*; *Δilv1*; *Δadh1*; *Δgpd1*); JWY18 (*∆ilv2*; *Δbdh1*; *Δbdh2*; *Δleu4*; *Δleu9*; *Δecm31*; *Δilv1*; *Δadh1*; *Δgpd2*); JWY19 (*∆ilv2*; *Δbdh1*; *Δbdh2*; *Δleu4*; *Δleu9*; *Δecm31*; *Δilv1*; *Δadh1*; *Δgpd1*; *Δgpd2*); JWY23 (*∆ilv2*; *Δbdh1*; *Δbdh2*; *Δleu4*; *Δleu9*; *Δecm31*; *Δilv1*; *Δadh1*; *Δgpd1*; *Δgpd2; Δald6*). **b** Isobutanol production. **c** Glycerol production. **d** Ethanol production

As the deletion of *PDC1* and *PDC5* had a strong impact on yeast physiology completely preventing synthesis of ethanol and isobutanol, and single deletions did not reduce ethanol formation significantly, we thought of disrupting ethanol formation by deletion of the *ADH1* gene, encoding the major alcohol dehydrogenase, in strain JWY04, resulting in strain JWY16. Deletion of *ADH1* resulted in an immensely decreased ethanol production down to 2.27 g/L compared to JWY04 (13.42 g/L) (Fig. 7d). However, isobutanol production was nearly not affected (0.52 g/L after 28 h) (Fig. 7b). Instead, glycerol titers increased massively from 2.91 g/L (after 24 h) in JWY04 to 10.96 g/L (after 72 h) in strain JWY16 (Fig. 7c). We hypothesized that this resulted from the fact that usually a considerable share of NADH is regenerated to NAD^+^ via ethanol formation by reduction of acetaldehyde to ethanol catalyzed by Adh1. To compensate the loss of NAD^+^ regeneration capacity in Adh1-deficient cells, glycerol formation is increased in strain JWY16. But although glucose was still available (Fig. 7a), the glycerol titer approached its maximum already after 48 h. Moreover, isobutanol production stopped at a similar time point when the glycerol concentration approached its maximum. These results indicate that either the capacity of the cytosolic isobutanol pathway is not high enough and cannot compete with the ethanol and glycerol biosynthesis pathways, or this might also indicate a redox cofactor imbalance in the isobutanol pathway, as only one of the two glycolytic NADH molecules can be reoxidized via isobutyraldehyde reduction. The other redox reaction, catalyzed by Ilv5, is specific for NADPH, and *S. cerevisiae* cells do not have transhydrogenase activity for converting NADPH into NADH [2].

To explore the role of glycerol biosynthesis on NAD^+^/NADH cofactor regeneration in more detail, we suppressed additionally glycerol biosynthesis in the Adh1-deficient cells. While glycerol biosynthesis was only slightly reduced by deleting glycerol-3-phosphate dehydrogenase 1 (*GPD1*) and *GPD2* separately (strains JWY17 and JWY18, respectively), glycerol biosynthesis was nearly completely blocked by deleting *GPD1* and *GPD2* together (strain JWY19). Fermentation experiments showed that the deletion of *GPD1* showed almost the same maximum titers of glycerol (11.04 g/L) (Fig. 7c) and isobutanol (0.43 g/L) (Fig. 7b). In contrast, the Gpd2-deficient strain JWY18 showed a distinct decrease of glycerol synthesis (9.14 g/L after 72 h) and a strong increase of the isobutanol titer up to 1.02 g/L (25.41 mg/g glucose) after 120 h. Moreover, double deletion of *GPD1* and *GPD2* in strain JWY19 abolished glycerol synthesis almost completely (0.15 g/L after 120 h) and the isobutanol level was even further increased showing a maximum isobutanol titer of 1.32 g/L (40.51 mg/g glucose) after 120 h.

As Ida et al. [21] and Milne et al. [28] have shown that also isobutyric acid production from isobutyraldehyde competes with isobutanol synthesis, the *ALD6* gene encoding one of the major aldehyde dehydrogenases in yeast was additionally deleted in strain JWY19, resulting in strain JWY23. After the deletion of Ald6, the isobutyric acid titer decreased from 0.22 g/L (strain JWY19) to below 0.04 g/L (strain JWY23). The *ald6* deletion accelerated glucose consumption and isobutanol formation and further increased isobutanol production up to a titer of 2.09 g/L after 96 h (Fig. 7b) with a yield of 59.55 mg/g glucose in JWY23. Compared to JWY19, the Ald6-deficient cells consumed glucose almost completely after 120 h (1.39 g/L) (Fig. 7a) and reached the highest isobutanol titers already after about 96 h. As strain JWY19 obviously had not reached maximal isobutanol titers even after 120 h, this might also explain the difference between the measured decrease in isobutyrate synthesis and increase in isobutanol production.

## Conclusion

Isobutanol is a promising biofuel, but its native biosynthesis level in yeast *S. cerevisiae* is very low. The current maximum isobutanol production with *S. cerevisiae* is still far below the theoretical yields of 410 mg/g glucose [13]. In this study, by successively blocking 2,3-butanediol, pantothenate, leucine and isoleucine biosynthesis pathways, we could successfully inhibit non-essential isobutanol competing pathways and by this optimize and increase the metabolic flux toward isobutanol synthesis in *S. cerevisiae* strain JWY04. In combination with the overexpression of the enzymes of valine biosynthesis in the cytosol, we could achieve a 57-fold increased isobutanol production of 0.56 g/L (13.54 mg/g glucose) compared to the parental strain CEN.PK113-7D. A 136-fold increase of the isobutanol production to a titer of 1.32 g/L (40.51 mg/g glucose) was achieved in strain JWY19 by additional inhibition of alternative pyruvate-consuming and NAD^+^-regenerating reactions of the ethanol and glycerol biosynthesis pathways to force regeneration via the isobutanol-producing Ehrlich pathway. Finally, additional reduction of isobutyric acid by-product formation resulted in a more than 200-fold increase of isobutanol production of up to 2.09 g/L with a yield of 59.55 mg/g glucose. This yield is one of the highest ever obtained for *S. cerevisiae* and is in the same range as those reported by Zhao et al. [37] who used a mitochondrial isobutanol pathway and introduced optogenetic circuits to shift cells from a light-induced growth phase to a darkness-induced isobutanol production phase.

The results indicate that the capacity of the isobutanol pathways cannot yet compete with the ethanol and glycerol biosynthesis pathways. Moreover, also redox cofactor imbalances—generation of NADH in glycolysis, NADPH utilization by Ilv5, NADH/NADPH utilization by Adhs—seem to contribute to the still limited formation of isobutanol. In accordance with Matsuda et al. [27], we hypothesize that besides a further optimization of the metabolic flux into and through the isobutanol pathway [33], balanced and precisely regulated levels of the redox cofactors NADH and NADPH are also important factors for isobutanol synthesis and should be considered to further increase isobutanol production.

## Methods

### Cultivations of microorganisms

*Saccharomyces cerevisiae* strains used in this study were derived from CEN.PK113-7D (*MATa URA3 HIS3 LEU2 TRP1 MAL2*-*8c SUC2*). 1 mL glycerol (25% v/v) stocks were prepared from exponential growing cells and frozen for storage at − 80 °C. For strain construction works, cells were grown aerobically in YEP medium (20 g/L bacteriological peptone, 10 g/L yeast extract). The carbon source glucose was autoclaved separately and added subsequently to a final concentration of 2% (w/v). Ethanol as carbon source was also added afterward to the medium to a final concentration of 2% (v/v). For fermentations, pre-cultures and fermentation cultures were grown aerobically in shake flasks in synthetic complete media (SC) (1.7 g/L yeast nitrogen base without amino acids, 5 g/L ammonium sulfate) supplemented with amino acids lacking valine as described previously [3]. Synthetic media were adjusted to pH 6.3 with potassium hydroxide. As carbon source, glucose was autoclaved separately and added to a final concentration of 2% (w/v) for pre-cultures and 4% (w/v) for fermentation cultures, respectively. If necessary, G418 (200 µg/mL), clonNAT/nourseothricin (100 µg/mL) and/or hygromycin B (200 µg/mL) were added for selection of *kanMX*, *natNT2* and/or *hphNT1* markers to the medium, respectively.

For fermentation experiments, cells of pre-cultures were harvested in exponential phase (OD_600nm_ ≤ 3), washed and incubated in a final volume of 50 mL in 100 mL shake flasks at an OD_600nm_ of 8 at 30 °C. Samples were taken at different time points for OD_600nm_ analyses and metabolic measurements via high-performance liquid chromatography (HPLC).

*Escherichia coli* DH10β was used and grown in lysogeny broth (LB) at 37 °C with 100 µg/mL ampicillin for plasmid selection.

### Growth and metabolite analyses via HPLC

To observe cell growth during fermentations, the cell density of the collected samples (OD_600nm_) was measured via spectrophotometer (Ultraspec 2100 pro spectrophotometer, GE Healthcare, USA). For metabolite analyses via HPLC, samples were centrifuged at 16,000×*g* for 5 min and 450 µL of supernatant was mixed and vortexed with 50 µL 5-sulfosalicylic acid for precipitation of proteins. After an additional centrifugation step (16,000×*g* for 5 min), the supernatant was analyzed via HPLC. The HPLC (Thermo Fisher, Germany) was equipped with a HyperREZ XP Carbohydrate H + column (300 × 700 mm, 8 micron; Thermo Fisher Scientific, Germany), coupled to a refractive index detector (Shodes RI-101, Shoko Scientific Co., Kanagawa, Japan). 5 mM of H_2_SO_4_ was used as mobile phase with a constant flow rate of 0.6 mL/min. The column temperature was kept constant at 65 °C [17].

### Plasmid and strain construction

Plasmids were assembled via Gibson assembly [14] or in vivo homologous recombination in yeast [30] (Table 1).

**Table 1:** List of plasmids used in this work

Plasmid name	Marker	Description	References
pRCC-K	*kanMX*	Rox3p-cas9-CYC1t; SNR52p-gRNA-SUP4t	[12]
pRCC-N	*natMX*	As pRCC-K, but with *natMX* resistance marker	[12]
pRCC-K_Bdh1/2	*kanMX*	pRCC-K with target sequence (GAAAATCTATGTACCCACGC) for *bdh1/2* deletion	This work
pRCC-N_Leu4/9	*natMX*	pRCC-N with target sequence (TGTCACAATGACCGTGGTTG) for *leu4/9* deletion	This work
pRCC-K_Ecm31	*kanMX*	pRCC-K with target sequence (GAAGAACTGTGCTCCCG) for *ecm31* deletion	This work
pRCC-K_Ilv1	*kanMX*	pRCC-K with target sequence (TACTTTACCCGACGTCCC) for *ilv1* deletion	This work
pRCC-K_Bat1	*kanMX*	pRCC-K with target sequence (ACAAGAGCTTGGCCAGG) for *bat1* deletion	This work
pRCC-K_Bat2	*kanMX*	pRCC-K with target sequence (ACAAGAGCTTGGCCAGG) for *bat2* deletion	This work
pRCC-K_Pdc1	*kanMX*	pRCC-K with target sequence (TGTTCCAGACACGACGTCA) for *pdc1* deletion	This work
pRCC-K_Pdc5	*kanMX*	pRCC-K with target sequence (ACGAAGTAACCTCACAATC) for *pdc5* deletion	This work
pRCC-K_Mth1	*kanMX*	pRCC-K with target sequence (GCAGTATGCATTCAGCGAGC) for Mth1 modification	This work
pRCC-K_Adh1	*kanMX*	pRCC-K with target sequence (TAACTTGATGGCCGGTCACT) for *adh1* deletion	This work
pRCC-K_Gpd1	*kanMX*	pRCC-K with target sequence (GTTTCGTCGAAGGTCTAGGC) for *gpd1* deletion	Boles lab stock
pRCC-N_Gpd2	*natMX*	pRCC-K with target sequence (CCCTTACATGAGGGGCCACG) for *gpd2* deletion	This work
pRCC-K_Ald6	*kanMX*	pRCC-K with target sequence (AAAACTTTGGCCTTAGCCCG) for *ald6* deletion	This work
IsoV100 (p425-synthILV235)	*kanMX*	2μ-plasmid with integrative ILV cassette which contains truncated ORFs of codon-optimized *ILV2∆N54*, codon-optimized *ILV5∆N48* and codon-optimized *ILV3∆N19* of *S. cerevisiae*; codon-optimized *ILV2∆N54* under control of shortened *HXT7* promoter and *CYC1* terminator, codon-optimized *ILV5∆N48* under control of *FBA1* promoter and *PGK1* terminator, codon-optimized *ILV3∆N19* under control of *PFK1* promoter and *FBA1* terminator, *loxP*-*kanMX*-*loxP* resistance gene, flanked at 369 bp and 385 bp homologous to *FMO1* locus, respectively, *LEU2* marker gene; capability of integration into chromosomeVIII of codon-optimized ILV-cassette through in vivo recombination after restriction by *Asc*I/*Pac*I In this work, p425-synthILV235 (IsoV100) was only used as an episomal 2µ-plasmid with *kanMX* (G418) as the selectable marker	[3]
pRS62N	*natMX*	*2µ*, *natNT2*, *AmpR*, shortened *HXT7* promotor (p_*HXT7*_^− *1*− *392*^) and *CYC1* terminator	[9]
pRS62N-IlvC6E6	*natMX*	pRS62N with IlvC^6E6^ from *E. coli*	This work

### CRISPR–Cas9 mediated deletions and integrations

Genomic engineering of yeast strains originating from CEN.PK113-7D were performed by using the CRISPR–Cas9 technique as described by Generoso et al. [12]. Transformations of CRISPR–Cas9 vectors with pRCC-K or pRCC-N backgrounds for deletions were performed following Gietz and Schiestl [15]. Transformants were selected and gene deletions verified by colony PCR. Finally, the defined clones were cured from CRISPR–Cas9 vectors.

### List of strains

See Tables 2 and 3.

**Table 2:** Yeast strains obtained for this work

Strain	Organism	Genotype	Description
CEN.PK113-7D	*S. cerevisiae*	*MATa*; *MAL2*-*8c*; *SUC2*	Euroscarf, Germany

**Table 3:** Yeast strains created in this work, originating from CEN.PK113-7D

Strain	Modifications
JWY0	*Δilv2*
JWY01	*Δilv2*; *Δbdh1*; *Δbdh2*
JWY02	*Δilv2*; *Δbdh1*; *Δbdh2*; *Δleu4*; *Δleu9*
JWY03	*Δilv2*; *Δbdh1*; *Δbdh2*; *Δleu4*; *Δleu9*; *Δecm31*
JWY04	*Δilv2*; *Δbdh1*; *Δbdh2*; *Δleu4*; *Δleu9*; *Δecm31*; *Δilv1*
JWY05	*Δilv2*; *Δbdh1*; *Δbdh2*; *Δleu4*; *Δleu9*; *Δecm31*; *Δilv1*; *Δbat1*
JWY06	*Δilv2*; *Δbdh1*; *Δbdh2*; *Δleu4*; *Δleu9*; *Δecm31*; *Δilv1*; *Δbat2*
JWY07	*Δilv2*; *Δbdh1*; *Δbdh2*; *Δleu4*; *Δleu9*; *Δecm31*; *Δilv1*; *Δbat1*; *Δbat2*
JWY12	*Δilv2*; *Δbdh1*; *Δbdh2*; *Δleu4*; *Δleu9*; *Δecm31*; *Δilv1*; *Δpdc1*::*MTH1*; *Δpdc5*
JWY13	*Δilv2*; *Δbdh1*; *Δbdh2*; *Δleu4*; *Δleu9*; *Δecm31*; *Δilv1*; *Δpdc1*; *Δpdc5*; *Δmth1*(+169; +393)
JWY14	*Δilv2*; *Δbdh1*; *Δbdh2*; *Δleu4*; *Δleu9*; *Δecm31*; *Δilv1*; *Δpdc1*
JWY15	*Δilv2*; *Δbdh1*; *Δbdh2*; *Δleu4*; *Δleu9*; *Δecm31*; *Δilv1*; *Δpdc5*
JWY16	*Δilv2*; *Δbdh1*; *Δbdh2*; *Δleu4*; *Δleu9*; *Δecm31*; *Δilv1*; *Δadh1*
JWY17	*Δilv2*; *Δbdh1*; *Δbdh2*; *Δleu4*; *Δleu9*; *Δecm31*; *Δilv1*; *Δadh1*; *Δgpd1*
JWY18	*Δilv2*; *Δbdh1*; *Δbdh2*; *Δleu4*; *Δleu9*; *Δecm31*; *Δilv1*; *Δadh1*; *Δgpd2*
JWY19	*Δilv2*; *Δbdh1*; *Δbdh2*; *Δleu4*; *Δleu9*; *Δecm31*; *Δilv1*; *Δadh1*; *Δgpd1*; *Δgpd2*
JWY23	*Δilv2*; *Δbdh1*; *Δbdh2*; *Δleu4*; *Δleu9*; *Δecm31*; *Δilv1*; *Δadh1*; *Δgpd1*; *Δgpd2; Δald6*

### List of primers

See Table 4.

**Table 4:** List of primers used in this work

Primer name	Sequence	Explanation
pRCC2_Fw	TGTTGTCTGACATTTTGAGAGTTAACACCGAAATTACCAAGGCTC	Primer for pRCCK/pRCCN amplification
pRCC1_Rv	CTTGGTGGTGTTCGTCGTATCTCTTAATCATAGAAGCAGACAATGGAG	Primer for pRCCK/pRCCN amplification
CC-Bdh1_Fw	GAAAATCTATGTACCCACGCGTTTTAGAGCTAGAAATAGCAAGTTAAA ATAAGG	*BDH1* target for pRCCK
CC-Bdh1_Rv	GCGTGGGTACATAGATTTTCGATCATTTATCTTTCACTGCGGAG	*BDH1* target for pRCCK
JWP001-DR-BDH1/2	GCAATAAGAATAACAATAAATTCATTGAACATATTTCAGATGACAAAAT AATATTTGGGGCCCCTCGCGGCTCATTTGTA	Donor DNA for *∆bdh1*
JWP002-DR-BDH1/2c	TACAAATGAGCCGCGAGGGGCCCCAAATATTATTTTGTCATCTGAAAT ATGTTCAATGAATTTATTGTTATTCTTATTGC	Donor DNA for *∆bdh1*
A1-Bdh2	TGACTGTGTTTGTGGTTCTC	PCR control of *∆bdh1* and *∆bdh2*
A4-Bdh1	TCGTCTTTGTTCCCACATTC	PCR control of *∆bdh1* and *∆bdh2*
CC-Leu4-9_Fw	ACAATGACCGTGGTTGGTTTTAGAGCTAGAAATAGCAAGTTAAAATA AGG	*LEU4/9* target for pRCCK
CC-Leu4-9_Rv	CAACCACGGTCATTGTGACAGATCATTTATCTTTCACTGCGGAG	*LEU4/9* target for pRCCK
DR-Leu4	TACTGTAGACTTTTTCCTTACAAAAAGACAAGGAACAATCGAACTTTTC TGTATTTCAGGACTTATTCGCTTCTATTTAT	Donor DNA for *∆leu4*
JWP003-DR-Leu4c	ATAAATAGAAGCGAATAAGTCCTGAAATACAGAAAAGTTCGATTGTTC CTTGTCTTTTTGTAAGGAAAAAGTCTACAGTA	Donor DNA for *∆leu4*
A1-Leu4	TTGTACAGTAACGGCCAGTC	PCR control of *∆leu4*
A4-Leu4	TTCGTCACTAACCGCCAAAC	PCR control of *∆leu4*
DR-Leu9	GGATAATACTATCGGCACATTATCATTTAGCCGCGTAGCCTAGAAAGG AGTAGCTTATGATTACTCATGTTATATATATA	Donor DNA for *∆leu9*
JWP004-DR-Leu9c	TATATATATAACATGAGTAATCATAAGCTACTCCTTTCTAGGCTACGCG GCTAAATGATAATGTGCCGATAGTATTATCC	Donor DNA for *∆leu9*
A1-Leu9	GGTAACGGTCGTAGTGAATG	PCR control of *∆leu9*
A4-Leu9	TGTTCTCCCTTCACAAAGTC	PCR control of *∆leu9*
CC-Ecm31_Fw	GAAGAACTGTGCTCCCGGTTTTAGAGCTAGAAATAGCAAGTTAAAATAA GG	*ECM31* target for pRCCN
CC-Ecm31_Rv	GGAGCACAGTTCTTCAATGATCATTTATCTTTCACTGCGGAG	*ECM31* target for pRCCN
DR-Ecm31	ATTAGCTTGCCATAAAATTAGGGAAATTTTTACTCACAATAATATATAGA TAAAAATCACTGCATAGGGAAAAAAACTTT	Donor DNA for *∆ecm31*
JWP005_DR-Ecm31c	AAAGTTTTTTTCCCTATGCAGTGATTTTTATCTATATATTATTGTGAGTA AAAATTTCCCTAATTTTATGGCAAGCTAAT	Donor DNA for *∆ecm31*
A1-Ecm31	ATGTACACGACAGACATTCC	PCR control of *∆ecm31*
A4-Ecm31	TATTATAAAGCGGCCAGCTC	PCR control of *∆ecm31*
CC-Ilv1_Fw	TACTTTACCCGACGTCCCGTTTTAGAGCTAGAAATAGCAAGTTAAAAT AAGG	*ILV1* target for pRCCK
CC-Ilv1_Rv	GACGTCGGGTAAAGTAACGATCATTTATCTTTCACTGCGGAG	*ILV1* target for pRCCK
DR-Ilv1	CAAGCCACATTTAAACTAAGTCAATTACACAAAGTTAGTGAACCGACA ATTTACTTTATAAATTTACGCAACAACTTGTT	Donor DNA for *∆ilv1*
JWP006-DR-ilv1c	AACAAGTTGTTGCGTAAATTTATAAAGTAAATTGTCGGTTCACTAACT TTGTGTAATTGACTTAGTTTAAATGTGGCTTG	Donor DNA for *∆ilv1*
A1-Ilv1	AATTCACTAGCGGCTCCTTG	PCR control of *∆ilv1*
A4-Ilv1	ATGGCTATGTGGAAGAAGTC	PCR control of *∆ilv1*
CC-Bat12_Fw	ACAAGAGCTTGGCCAGGGTTTTAGAGCTAGAAATAGCAAGTTAAAA TAAGG	*BAT1* and *BAT2* target for pRCCK
CC-Bat1_Rv	CCTGGCCAAGCTCTTGTAGCGATCATTTATCTTTCACTGCGGAG	*BAT1* target for pRCCK
CC-Bat2_Rv	CCTGGCCAAGCTCTTGTGGCGATCATTTATCTTTCACTGCGGAG	*BAT2* target for pRCCK
DR-Bat1	TATAAACGCAAAATCAGCTAGAACCTTAGCATACTAAAACTGATAA TGAAGGTAAACATCCCCTCCCCCCCCAAAAAAAA	Donor DNA for *∆bat1*
JWP007-DR-Bat1c	TTTTTTTTGGGGGGGGAGGGGATGTTTACCTTCATTATCAGTTTTAG TATGCTAAGGTTCTAGCTGATTTTGCGTTTATA	Donor DNA for *∆bat1*
A1-Bat1	TTTAATGGCCCATCCGATCC	PCR control of *∆bat1*
A4-Bat1	AAGTCCAGCGAGATACCTTG	PCR control of *∆bat1*
DR-Bat2	AAATTTAAGGGAAAGCATCTCCACGAGTTTTAAGAACGATAGTATCG CTATTGCTACGTAAAGTAATTAAAAGTTAAAAA	Donor DNA for *∆bat2*
JWP008-DR-Bat2c	TTTTTAACTTTTAATTACTTTACGTAGCAATAGCGATACTATCGTTCT TAAAACTCGTGGAGATGCTTTCCCTTAAATTT	Donor DNA for *∆bat2*
A1-Bat2	GTGAGAGGAGATCCGAAATGAG	PCR control of *∆bat2*
A4-Bat2	TCCACCGACATTACGGAAAC	PCR control of *∆bat2*
JWP051-CC-Pdc1_Fw(4)	TGTTCCAGACACGACGTCAGTTTTAGAGCTAGAAATAGCAAGTTAA AATAAGG	*PDC1* target for pRCCK
JWP052-CC-Pdc1_Rv(4)	CTGACGTCGTGTCTGGAACAGATCATTTATCTTTCACTGCGGAG	*PDC1* target for pRCCK
DR_PDC1-Fw	TCTCAATTATTATCTTCTACTCATAACCTCACGCAAAATAACACAGT CAAATCAATCAAAGCGATTTAATCTCTAATTATTAGTTAAAGTTTTA TAAGCATTTTTATGTAACGAAAAATA	Donor DNA for *∆pdc1*
DR_PDC1-Rv	TATTTTTCGTTACATAAAAATGCTTATAAAACTTTAACTAATAATTA GAGATTAAATCGCTTTGATTGATTTGACTGTGTTATTTTGCGTGAG GTTATGAGTAGAAGATAATAATTGAGA	Donor DNA for *∆pdc1*
A1-PDC1	GAAATCAGCTTGTGGGTATTGTTCAGAG	PCR control of *∆pdc1*
A4-PDC1	CCTGGTGGCATTTGCAAAATG	PCR control of *∆pdc1*
JWP021-CC-Pdc5_Fw	ACGAAGTAACCTCACAATCGTTTTAGAGCTAGAAATAGCAAGT TAAAATAAGG	*PDC5* target for pRCCK
JWP022-CC-Pdc5_Rv	CGATTGTGAGGTTACTTCGTGATCATTTATCTTTCACTGCGGAG	*PDC5* target for pRCCK
DR_PDC5-Fw	ACTTATTTCACATAATCAATCTCAAAGAGAACAACACAATACAATAA CAAGAAGAACAAAGCTAATTAACATAAAACTCATGATTCAACGTTT GTGTATTTTTTTACTTTTGAAGGTTAT	Donor DNA for *∆pdc5*
DR_PDC5-Rv	ATAACCTTCAAAAGTAAAAAAATACACAAACGTTGAATCATGAGTTT TATGTTAATTAGCTTTGTTCTTCTTGTTATTGTATTGTGTTGTTCTCTT TGAGATTGATTATGTGAAATAAGT	Donor DNA for *∆pdc5*
A1-PDC5	CGTATACGAATTCCTTCAACAAAGGCC	PCR control of *∆pdc5*
A4-PDC5	TAAGAAGGCATGTTGGCCTCTGTTTC	PCR control of *∆pdc5*
JWP023-Mth1/Pdc1_Fw	TGCTTATAAAACTTTAACTAATAATTAGAGATTAAATCGCATGTTTG TTTCACCACCACCAG	Primer for synthesis of Donor DNA of Mth1 wt
JWP024-Mth1/Pdc1_Rv	TCATAACCTCACGCAAAATAACACAGTCAAATCAATCAAATCAGGA TACTGAATCCGGCTGC	Primer for synthesis of Donor DNA of Mth1 wt
JWP025-Mth1-∆T_Rv	CATTAGTTAGTTGCGTGTGCACAGTAGAGGGGGCAGAAAACATTG ATAGTGGCAAACTTTG	Primer for synthesis of Donor DNA of Mth1∆T
JWP026-Mth1-∆T_Fw	CAGTGATAATGCTTCTTTTCAAAGTTTGCCACTATCAATGTTTTCTGC CCCCTCTACTGTG	Primer for synthesis of Donor DNA of Mth1∆T
JWP043-CC-MTH1_Fw	GCAGTATGCATTCAGCGAGCGTTTTAGAGCTAGAAATAGCAAGT TAAAATAAGG	*MTH1* target for pRCCK
JWP044-CC-MTH1_Rv	CGCTCGCTGAATGCATACTGCGATCATTTATCTTTCACTGCGGAG	*MTH1* target for pRCCK
JWP045-DR-MTH1dT_Fw	CAGTGATAATGCTTCTTTTCAAAGTTTGCCACTATCAATGTTTTCTGCCCC CTCTACTGTGCACACGCAACTAACTAATG	Donor DNA for *MTH1∆T* modification
JWP046-DR-MTH1dT_Rv	CATTAGTTAGTTGCGTGTGCACAGTAGAGGGGGCAGAAAACATTGATAG TGGCAAACTTTGAAAAGAAGCATTATCACTG	Donor DNA for *MTH1∆T* modification
JWP047-MTH1_Fw	ATGTTTGTTTCACCACCACCAGC	PCR control of *MTH1* modifications
JWP048-MTH1_Rv	TCAGGATACTGAATCCGGCTGC	PCR control of *MTH1* modifications
JWP053-CC-Adh1_Fw	TAACTTGATGGCCGGTCACTGTTTTAGAGCTAGAAATAGCAAGTT AAAATAAGG	*ADH1* target for pRCCK
JWP054-CC-Adh1_Rv	CAGTGACCGGCCATCAAGTTAGATCATTTATCTTTCACTGCGGAG	*ADH1* target for pRCCK
JWP055-DR-Adh1_Fw	TCAAGCTATACCAAGCATACAATCAACTATCTCATATACAGCGAAT TTCTTATGATTTATGATTTTTATTATTAAATAAG	Donor DNA for *∆adh1*
JWP056-DR-Adh1_Rv	CTTATTTAATAATAAAAATCATAAATCATAAGAAATTCGCTGTATA TGAGATAGTTGATTGTATGCTTGGTATAGCTTGA	Donor DNA for *∆adh1*
Sbp26-Chk_DelADH1_A1	GCAACCAAACCCATACATCG	PCR control of *∆adh1*
Sbp27-Chk_DelADH1_A4	GGGCGGAGCGTTCTAATTG	PCR control of *∆adh1*
vsp381_CC-GPD1_rev	GCCTAGACCTTCGACGAAACGATCATTTATCTTTCACTGCGGAG	*GPD1* target for pRCCK
vsp382_CC-GPD1_fw	GTTTCGTCGAAGGTCTAGGCGTTTTAGAGCTAGAAATAGCAAGTT AAAATAAGG	*GPD1* target for pRCCK
vsp383_CC-GPD1-Donor	CACGTAGACTGGCTTGGTATTGGCAGTTTCGTAGTTATATATTTAT TGGAGAAAGATAACATATCATACTTTCCCCCACT	Donor DNA for *∆gpd1*
JWP058-DR-Gpd1_Rv	AGTGGGGGAAAGTATGATATGTTATCTTTCTCCAATAAATATATA ACTACGAAACTGCCAATACCAAGCCAGTCTACGTG	Donor DNA for *∆gpd1*
vsp384_pGPD1_fw	GTACAGCTGATGGGACCTTGCCG	PCR control of *∆gpd1*
vsp385_tGPD1_rev	GCTCCGTATTATCTTCGTCGTGGGG	PCR control of *∆gpd1*
JWP059-CC-Gpd2_Fw	CCCTTACATGAGGGGCCACGGTTTTAGAGCTAGAAATAGCAAGTT AAAATAAGG	*GPD2* target for pRCCK
JWP060-CC-Gpd2_Rv	CCGTGGCCCCTCATGTAAGGGGATCATTTATCTTTCACTGCGGAG	*GPD2* target for pRCCK
JWP061-DR-Gpd2_Fw	CTCTTTCCCTTTCCTTTTCCTTCGCTCCCCTTCCTTATCAACACTCTCC CCCCCCCTCCCCCTCTGATCTTTCCTGTTGC	Donor DNA for *∆gpd2*
JWP062-DR-Gpd2_Rv	GCAACAGGAAAGATCAGAGGGGGAGGGGGGGGGAGAGTGTTGA TAAGGAAGGGGAGCGAAGGAAAAGGAAAGGGAAAGAG	Donor DNA for *∆gpd2*
vsp269_pGPD2_fw	GGAACATCCGAGCACCCGCGCC	PCR control of *∆gpd2*
vsp270_tGPD2_rev	GGCGGCATCGAAATCTTCTTCTTGCCC	PCR control of *∆gpd2*
Vsp388_CC-ALD6_fw	AAAACTTTGGCCTTAGCCCGGTTTTAGAGCTAGAAATAGCAAGTTAAAATAAGG	*ALD6* target for pRCCK
vsp389_CC-ALD6_rev	CGGGCTAAGGCCAAAGTTTTGATCATTTATCTTTCACTGCGGAG	*ALD6* target for pRCCK
vsp390_CC-ALD6-Donor	AACATCTTTAACATACACAAACACATACTATCAGAATACATGTACCAACCTGCATTTCTTTCCGTCATATACACAAAATA	Donor DNA for *∆ald6*
JWY092_DR_Ald6_c	TATTTTGTGTATATGACGGAAAGAAATGCAGGTTGGTACATGTATTCTGATAGTATGTGTTTGTGTATGTTAAAGATGTT	Donor DNA for *∆ald6*
vsp393_ALD6_rev	TACCGGCCTTCAACATCTTGGCC	PCR control of *∆ald6*
vsp394_ALD6_fw	TCCACGACACTGAATGGGCTACCC	PCR control of *∆ald6*

## Data Availability

The datasets used and/or analyzed during the current study are available from the corresponding author upon reasonable request.
